# Bis(2,2′-bipyridine-κ^2^ *N*,*N*′)bis­(dicyan­amido-κ*N* ^1^)cadmium

**DOI:** 10.1107/S1600536812044108

**Published:** 2012-10-31

**Authors:** Dasarath Mal, Rupam Sen, Paula Brandao, Zhi Lin

**Affiliations:** a Department of chemistry, CICECO, University of Aveiro, 3810-193 Portugal

## Abstract

In the title compound, [Cd(C_2_N_3_)_2_(C_10_H_8_N_2_)_2_], the Cd^II^ ion is coordinated in a distorted octa­hedral environment by four N atoms from two chelating 2,2′-bipyridine ligands and two N atoms from two monodentate dicyanamide ligands. The dihedral angle between the mean planes of the two bipyridine ligands is 87.67 (6)°.

## Related literature

For background to materials with metal–bpy–dca framework structures, see: Mal *et al.* (2006 ▶, 2007 ▶). For related structures, see: Wang *et al.* (2012 ▶); Luo *et al.* (2002 ▶). 
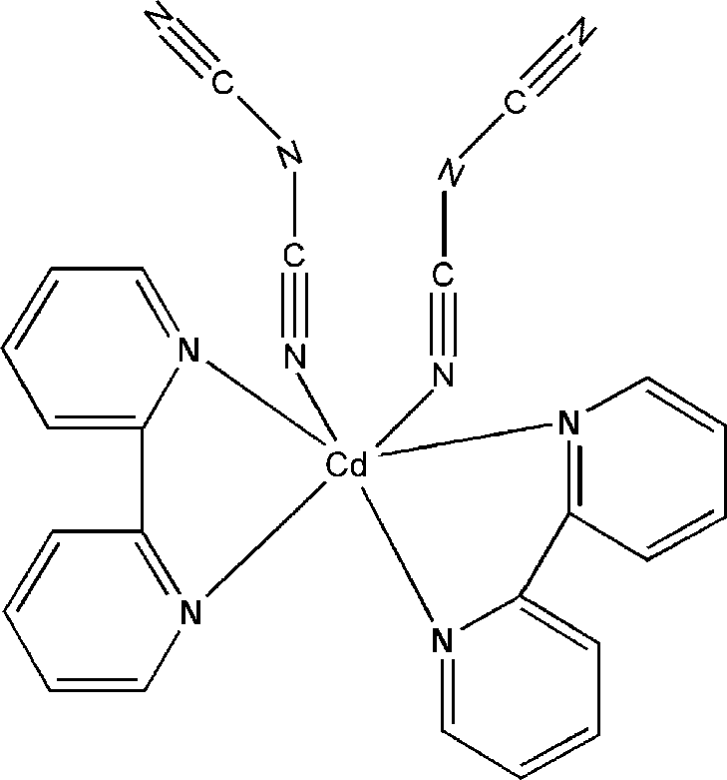


## Experimental

### 

#### Crystal data

[Cd(C_2_N_3_)_2_(C_10_H_8_N_2_)_2_]
*M* *_r_* = 556.87
Monoclinic, 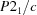

*a* = 9.5586 (3) Å
*b* = 14.9260 (5) Å
*c* = 16.7007 (6) Å
β = 100.521 (2)°
*V* = 2342.66 (14) Å^3^
*Z* = 4
Mo *K*α radiation
μ = 0.97 mm^−1^
*T* = 150 K
0.30 × 0.16 × 0.03 mm

#### Data collection

Bruker SMART CCD diffractometer
Absorption correction: multi-scan (*SADABS*; Bruker, 2008 ▶) *T*_min_ = 0.760, *T*_max_ = 0.972
25970 measured reflections
6309 independent reflections
5064 reflections with *I* > 2σ(*I*)
*R*_int_ = 0.033

#### Refinement

*R*[*F* ^2^ > 2σ(*F* ^2^)] = 0.028
*wR*(*F* ^2^) = 0.069
*S* = 1.01
6309 reflections
316 parameters
H-atom parameters constrained
Δρ_max_ = 0.48 e Å^−3^
Δρ_min_ = −0.39 e Å^−3^

### 

Data collection: *SMART* (Bruker, 2008 ▶); cell refinement: *SAINT* (Bruker, 2008 ▶); data reduction: *SAINT*; program(s) used to solve structure: *SHELXS97* (Sheldrick, 2008 ▶); program(s) used to refine structure: *SHELXL97* (Sheldrick, 2008 ▶); molecular graphics: *ORTEP-3* (Farrugia, 1997 ▶); software used to prepare material for publication: *SHELXL97* (Sheldrick, 2008 ▶).

## Supplementary Material

Crystal structure: contains datablock(s) I, global. DOI: 10.1107/S1600536812044108/lh5540sup1.cif

Structure factors: contains datablock(s) I. DOI: 10.1107/S1600536812044108/lh5540Isup2.hkl

Additional supplementary materials:  crystallographic information; 3D view; checkCIF report

## Figures and Tables

**Fig. 1.**
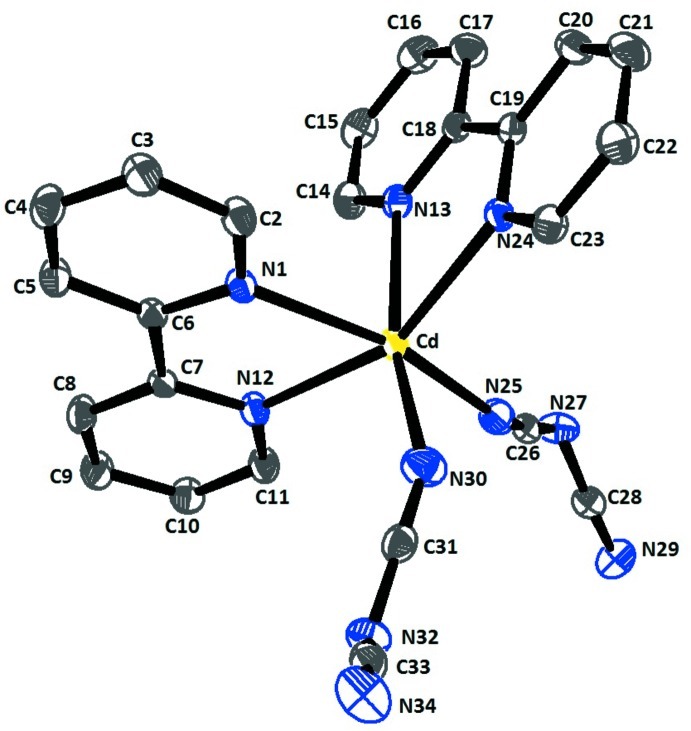
The molecular structure of the title complex with 30% displacement ellipsoids.

## supplementary crystallographic information

### Comment

Coordination polymers containing dicyanamide [dca^-^, N(CN)_2_^-^] have gained attention in the last decade due to their versatile binding modes where the three possible donor sites allow monodentate to pentadentate binding to the metal centre (Mal *et al.*, 2006; Wang *et al.*, 2012; Luo *et al.* 2002). Herein, we present the crystal structure of the title complex.

The molecular structure of the title compound is shown in Fig. 1. The Cd^II^ ion is coordinated by six N atoms, two of which are from monodentate dca ligands and four N atoms are from from two chelating bpy ligands. The coordination geometry is distorted octahedral. The Cd—N_bpy_ and Cd—N_dca_ bond distances are comparable with a previously reported cadmium-dca-bpy complex (Luo *et al.*, 2002). The Cd—N_dicyanamido_ bond lengths are slightly shorter than the Cd—N_bipyridine_ lengths. The crystal structure of the Mn(II) analog of the title compound has been published previously (Wang *et al.*, 2012).

### Experimental

An aqueous solution (5 ml) of dca (0.178 g, 2 mmol) was mixed with an aqueous solution (5 ml) of Cd(NO_3_)_2_.4H_2_O (0.155 g, 0.5 mmol), at room temperature. The solution was stirred for 10 min. Then a methanolic solution (8 ml) containing bpy (0.312 g, 2 mmol) was added drop wise into the above solution. After the mixture was stirred for about 15 minutes at room temperature. It was filtrated and the filtrate was left for slow evaporation in air. Plate-shaped colorless crystals of [Cd(N(CN)_2_)_2_(bpy)_2_] were obtained from the mother liquor by slow evaporation at room temperature after two weeks.

### Refinement

H atoms were placed in calculated positions with C—H = 0.95Å and were included in the refinement with U_iso_(H) = 1.2U_eq_(C).

### Crystal data

**Table:**

[Cd(C_2_N_3_)_2_(C_10_H_8_N_2_)_2_]	*F*(000) = 1112
*M**_r_* = 556.87	*D*_x_ = 1.579 Mg m^−^^3^
Monoclinic, *P*2_1_/*c*	Mo *K*α radiation, λ = 0.71073 Å
Hall symbol: -P 2ybc	Cell parameters from 320 reflections
*a* = 9.5586 (3) Å	θ = 3.0–29.2°
*b* = 14.9260 (5) Å	µ = 0.97 mm^−^^1^
*c* = 16.7007 (6) Å	*T* = 150 K
β = 100.521 (2)°	Plate, colourless
*V* = 2342.66 (14) Å^3^	0.30 × 0.16 × 0.03 mm
*Z* = 4	

### Data collection

**Table:**

Bruker SMART CCD diffractometer	6309 independent reflections
Radiation source: fine-focus sealed tube	5064 reflections with *I* > 2σ(*I*)
Graphite monochromator	*R*_int_ = 0.033
φ and ω scans	θ_max_ = 29.2°, θ_min_ = 1.8°
Absorption correction: multi-scan (*SADABS*; Bruker, 2008)	*h* = −12→13
*T*_min_ = 0.760, *T*_max_ = 0.972	*k* = −19→20
25970 measured reflections	*l* = −22→22

### Refinement

**Table:**

Refinement on *F*^2^	Primary atom site location: structure-invariant direct methods
Least-squares matrix: full	Secondary atom site location: difference Fourier map
*R*[*F*^2^ > 2σ(*F*^2^)] = 0.028	Hydrogen site location: inferred from neighbouring sites
*wR*(*F*^2^) = 0.069	H-atom parameters constrained
*S* = 1.01	*w* = 1/[σ^2^(*F*_o_^2^) + (0.0331*P*)^2^ + 0.4905*P*] where *P* = (*F*_o_^2^ + 2*F*_c_^2^)/3
6309 reflections	(Δ/σ)_max_ = 0.003
316 parameters	Δρ_max_ = 0.48 e Å^−^^3^
0 restraints	Δρ_min_ = −0.39 e Å^−^^3^

### Special details

**Table:**

Geometry. All e.s.d.'s (except the e.s.d. in the dihedral angle between two l.s. planes) are estimated using the full covariance matrix. The cell e.s.d.'s are taken into account individually in the estimation of e.s.d.'s in distances, angles and torsion angles; correlations between e.s.d.'s in cell parameters are only used when they are defined by crystal symmetry. An approximate (isotropic) treatment of cell e.s.d.'s is used for estimating e.s.d.'s involving l.s. planes.
Refinement. Refinement of *F*^2^ against ALL reflections. The weighted *R*-factor *wR* and goodness of fit *S* are based on *F*^2^, conventional *R*-factors *R* are based on *F*, with *F* set to zero for negative *F*^2^. The threshold expression of *F*^2^ > σ(*F*^2^) is used only for calculating *R*-factors(gt) *etc*. and is not relevant to the choice of reflections for refinement. *R*-factors based on *F*^2^ are statistically about twice as large as those based on *F*, and *R*- factors based on ALL data will be even larger.

### Fractional atomic coordinates and isotropic or equivalent isotropic displacement parameters (Å^2^)

**Table:**

	*x*	*y*	*z*	*U*_iso_*/*U*_eq_	
Cd	0.516014 (15)	0.704173 (9)	0.110929 (9)	0.02649 (5)	
N1	0.41014 (17)	0.82838 (11)	0.02936 (11)	0.0301 (4)	
C2	0.2810 (2)	0.86215 (15)	0.03317 (15)	0.0377 (5)	
H2	0.2321	0.8397	0.0736	0.045*	
C3	0.2167 (2)	0.92750 (15)	−0.01854 (16)	0.0427 (6)	
H3	0.1255	0.9499	−0.0139	0.051*	
C4	0.2872 (3)	0.95978 (15)	−0.07727 (17)	0.0458 (6)	
H4	0.2447	1.0044	−0.1145	0.055*	
C5	0.4210 (2)	0.92643 (14)	−0.08159 (15)	0.0402 (5)	
H5	0.4719	0.9485	−0.1213	0.048*	
C6	0.4796 (2)	0.86051 (12)	−0.02727 (12)	0.0278 (4)	
C7	0.6241 (2)	0.82153 (13)	−0.02755 (13)	0.0283 (4)	
C8	0.7115 (2)	0.85346 (15)	−0.07896 (15)	0.0407 (5)	
H8	0.6806	0.9009	−0.1159	0.049*	
C9	0.8445 (3)	0.81530 (16)	−0.07568 (18)	0.0502 (7)	
H9	0.9060	0.8366	−0.1102	0.060*	
C10	0.8865 (2)	0.74695 (16)	−0.02259 (17)	0.0447 (6)	
H10	0.9770	0.7196	−0.0200	0.054*	
C11	0.7949 (2)	0.71808 (14)	0.02755 (15)	0.0356 (5)	
H11	0.8244	0.6710	0.0651	0.043*	
N12	0.66591 (17)	0.75460 (11)	0.02463 (10)	0.0271 (3)	
N13	0.61503 (18)	0.80134 (11)	0.21846 (11)	0.0287 (4)	
C14	0.7419 (2)	0.84160 (15)	0.22300 (13)	0.0348 (5)	
H14	0.7974	0.8280	0.1828	0.042*	
C15	0.7948 (2)	0.90175 (16)	0.28339 (15)	0.0418 (5)	
H15	0.8852	0.9290	0.2852	0.050*	
C16	0.7134 (2)	0.92151 (16)	0.34129 (15)	0.0424 (6)	
H16	0.7470	0.9629	0.3836	0.051*	
C17	0.5827 (2)	0.88063 (14)	0.33726 (13)	0.0355 (5)	
H17	0.5255	0.8937	0.3767	0.043*	
C18	0.5361 (2)	0.82024 (12)	0.27498 (12)	0.0264 (4)	
C19	0.3954 (2)	0.77364 (12)	0.26621 (12)	0.0267 (4)	
C20	0.3047 (2)	0.78768 (14)	0.32058 (15)	0.0397 (5)	
H20	0.3319	0.8264	0.3659	0.048*	
C21	0.1738 (3)	0.74494 (16)	0.30853 (16)	0.0448 (6)	
H21	0.1094	0.7554	0.3447	0.054*	
C22	0.1381 (2)	0.68769 (15)	0.24420 (15)	0.0392 (5)	
H22	0.0499	0.6566	0.2354	0.047*	
C23	0.2340 (2)	0.67635 (15)	0.19240 (14)	0.0372 (5)	
H23	0.2093	0.6372	0.1472	0.045*	
N24	0.36027 (18)	0.71798 (11)	0.20291 (11)	0.0309 (4)	
N25	0.6584 (2)	0.59039 (14)	0.16670 (14)	0.0484 (5)	
C26	0.7001 (2)	0.54121 (14)	0.21884 (15)	0.0354 (5)	
N27	0.73104 (19)	0.48378 (13)	0.27762 (12)	0.0397 (4)	
C28	0.8637 (2)	0.46549 (14)	0.31152 (14)	0.0353 (5)	
N29	0.9733 (2)	0.44417 (17)	0.34649 (15)	0.0559 (6)	
N30	0.3634 (2)	0.61331 (13)	0.02748 (12)	0.0435 (5)	
C31	0.2583 (2)	0.59524 (13)	−0.01597 (14)	0.0343 (5)	
N32	0.1425 (2)	0.58623 (12)	−0.06940 (13)	0.0432 (5)	
C33	0.0734 (2)	0.51007 (15)	−0.08012 (14)	0.0344 (5)	
N34	0.00240 (19)	0.44904 (14)	−0.09561 (14)	0.0459 (5)	

### Atomic displacement parameters (Å^2^)

**Table:**

	*U*^11^	*U*^22^	*U*^33^	*U*^12^	*U*^13^	*U*^23^
Cd	0.02787 (8)	0.03095 (8)	0.02186 (8)	−0.00212 (6)	0.00773 (6)	−0.00153 (6)
N1	0.0289 (9)	0.0325 (8)	0.0294 (10)	0.0026 (7)	0.0065 (8)	−0.0025 (7)
C2	0.0318 (11)	0.0428 (12)	0.0401 (13)	0.0052 (9)	0.0111 (10)	−0.0044 (10)
C3	0.0324 (11)	0.0428 (12)	0.0510 (16)	0.0109 (10)	0.0020 (11)	−0.0049 (11)
C4	0.0445 (13)	0.0374 (12)	0.0509 (16)	0.0089 (10)	−0.0035 (12)	0.0056 (11)
C5	0.0443 (13)	0.0370 (11)	0.0388 (14)	0.0041 (10)	0.0062 (11)	0.0084 (10)
C6	0.0314 (10)	0.0264 (9)	0.0249 (10)	−0.0007 (8)	0.0031 (8)	−0.0048 (8)
C7	0.0321 (10)	0.0272 (9)	0.0269 (11)	−0.0009 (8)	0.0090 (9)	−0.0036 (8)
C8	0.0475 (13)	0.0372 (11)	0.0422 (14)	0.0033 (10)	0.0212 (11)	0.0083 (10)
C9	0.0491 (14)	0.0480 (14)	0.0626 (18)	0.0002 (11)	0.0347 (14)	0.0078 (12)
C10	0.0346 (12)	0.0457 (13)	0.0589 (17)	0.0044 (10)	0.0223 (12)	−0.0011 (12)
C11	0.0329 (11)	0.0353 (11)	0.0410 (13)	0.0065 (9)	0.0133 (10)	0.0006 (9)
N12	0.0288 (8)	0.0284 (8)	0.0255 (9)	0.0024 (7)	0.0088 (7)	−0.0001 (7)
N13	0.0280 (8)	0.0356 (9)	0.0227 (9)	−0.0041 (7)	0.0049 (7)	−0.0012 (7)
C14	0.0297 (11)	0.0466 (12)	0.0283 (12)	−0.0080 (9)	0.0057 (9)	−0.0051 (10)
C15	0.0361 (12)	0.0536 (13)	0.0350 (13)	−0.0155 (10)	0.0051 (10)	−0.0047 (11)
C16	0.0421 (13)	0.0481 (13)	0.0354 (14)	−0.0117 (10)	0.0034 (11)	−0.0138 (11)
C17	0.0369 (11)	0.0419 (11)	0.0283 (12)	−0.0028 (9)	0.0074 (9)	−0.0074 (9)
C18	0.0299 (10)	0.0275 (9)	0.0217 (10)	0.0009 (8)	0.0043 (8)	0.0017 (8)
C19	0.0301 (10)	0.0269 (9)	0.0239 (10)	0.0005 (8)	0.0072 (8)	0.0025 (8)
C20	0.0425 (13)	0.0434 (12)	0.0376 (13)	−0.0101 (10)	0.0188 (11)	−0.0136 (10)
C21	0.0454 (13)	0.0496 (13)	0.0473 (16)	−0.0104 (11)	0.0290 (12)	−0.0103 (12)
C22	0.0318 (11)	0.0450 (12)	0.0436 (14)	−0.0107 (9)	0.0145 (10)	−0.0046 (10)
C23	0.0367 (12)	0.0444 (11)	0.0326 (12)	−0.0116 (10)	0.0118 (10)	−0.0085 (10)
N24	0.0298 (9)	0.0382 (9)	0.0261 (9)	−0.0071 (7)	0.0087 (7)	−0.0058 (7)
N25	0.0481 (12)	0.0464 (11)	0.0526 (14)	0.0119 (9)	0.0146 (11)	0.0123 (10)
C26	0.0342 (11)	0.0325 (10)	0.0420 (14)	0.0008 (9)	0.0133 (10)	−0.0061 (10)
N27	0.0353 (10)	0.0417 (10)	0.0427 (12)	−0.0015 (8)	0.0083 (9)	0.0088 (9)
C28	0.0379 (12)	0.0388 (11)	0.0310 (12)	−0.0051 (9)	0.0110 (10)	−0.0014 (9)
N29	0.0376 (12)	0.0804 (16)	0.0485 (14)	0.0001 (11)	0.0051 (10)	0.0099 (13)
N30	0.0526 (12)	0.0437 (10)	0.0341 (11)	−0.0161 (10)	0.0079 (10)	−0.0037 (9)
C31	0.0452 (13)	0.0306 (10)	0.0307 (12)	−0.0054 (9)	0.0166 (11)	−0.0002 (9)
N32	0.0390 (11)	0.0396 (10)	0.0486 (13)	−0.0037 (8)	0.0019 (10)	0.0130 (9)
C33	0.0281 (10)	0.0406 (11)	0.0334 (12)	0.0036 (9)	0.0028 (9)	0.0059 (10)
N34	0.0316 (10)	0.0450 (11)	0.0561 (15)	0.0024 (9)	−0.0052 (10)	−0.0059 (10)

### Geometric parameters (Å, º)

**Table:**

Cd—N25	2.267 (2)	N13—C14	1.343 (3)
Cd—N30	2.273 (2)	C14—C15	1.376 (3)
Cd—N12	2.3347 (15)	C14—H14	0.9500
Cd—N24	2.3352 (16)	C15—C16	1.379 (3)
Cd—N13	2.3672 (17)	C15—H15	0.9500
Cd—N1	2.4111 (17)	C16—C17	1.381 (3)
N1—C6	1.339 (3)	C16—H16	0.9500
N1—C2	1.345 (3)	C17—C18	1.387 (3)
C2—C3	1.372 (3)	C17—H17	0.9500
C2—H2	0.9500	C18—C19	1.497 (3)
C3—C4	1.374 (4)	C19—N24	1.338 (3)
C3—H3	0.9500	C19—C20	1.381 (3)
C4—C5	1.387 (3)	C20—C21	1.387 (3)
C4—H4	0.9500	C20—H20	0.9500
C5—C6	1.385 (3)	C21—C22	1.366 (3)
C5—H5	0.9500	C21—H21	0.9500
C6—C7	1.500 (3)	C22—C23	1.381 (3)
C7—N12	1.338 (3)	C22—H22	0.9500
C7—C8	1.388 (3)	C23—N24	1.341 (3)
C8—C9	1.385 (3)	C23—H23	0.9500
C8—H8	0.9500	N25—C26	1.153 (3)
C9—C10	1.363 (4)	C26—N27	1.296 (3)
C9—H9	0.9500	N27—C28	1.320 (3)
C10—C11	1.386 (3)	C28—N29	1.147 (3)
C10—H10	0.9500	N30—C31	1.159 (3)
C11—N12	1.341 (3)	C31—N32	1.296 (3)
C11—H11	0.9500	N32—C33	1.311 (3)
N13—C18	1.342 (2)	C33—N34	1.137 (3)
			
N25—Cd—N30	94.29 (8)	C7—N12—C11	119.39 (17)
N25—Cd—N12	96.04 (6)	C7—N12—Cd	119.90 (12)
N30—Cd—N12	102.20 (6)	C11—N12—Cd	120.70 (14)
N25—Cd—N24	101.70 (7)	C18—N13—C14	118.97 (18)
N30—Cd—N24	92.35 (6)	C18—N13—Cd	117.65 (13)
N12—Cd—N24	156.12 (6)	C14—N13—Cd	123.28 (14)
N25—Cd—N13	91.17 (7)	N13—C14—C15	122.7 (2)
N30—Cd—N13	162.47 (6)	N13—C14—H14	118.6
N12—Cd—N13	93.75 (6)	C15—C14—H14	118.6
N24—Cd—N13	70.21 (6)	C14—C15—C16	118.3 (2)
N25—Cd—N1	165.14 (6)	C14—C15—H15	120.8
N30—Cd—N1	87.49 (7)	C16—C15—H15	120.8
N12—Cd—N1	69.20 (5)	C17—C16—C15	119.5 (2)
N24—Cd—N1	92.96 (6)	C17—C16—H16	120.2
N13—Cd—N1	91.49 (6)	C15—C16—H16	120.2
C6—N1—C2	118.44 (19)	C16—C17—C18	119.20 (19)
C6—N1—Cd	117.60 (13)	C16—C17—H17	120.4
C2—N1—Cd	123.74 (14)	C18—C17—H17	120.4
N1—C2—C3	123.1 (2)	N13—C18—C17	121.26 (18)
N1—C2—H2	118.4	N13—C18—C19	116.36 (17)
C3—C2—H2	118.4	C17—C18—C19	122.38 (18)
C2—C3—C4	118.5 (2)	N24—C19—C20	121.09 (19)
C2—C3—H3	120.8	N24—C19—C18	117.21 (17)
C4—C3—H3	120.8	C20—C19—C18	121.70 (19)
C3—C4—C5	119.2 (2)	C19—C20—C21	119.6 (2)
C3—C4—H4	120.4	C19—C20—H20	120.2
C5—C4—H4	120.4	C21—C20—H20	120.2
C6—C5—C4	119.2 (2)	C22—C21—C20	119.4 (2)
C6—C5—H5	120.4	C22—C21—H21	120.3
C4—C5—H5	120.4	C20—C21—H21	120.3
N1—C6—C5	121.58 (19)	C21—C22—C23	118.0 (2)
N1—C6—C7	116.02 (18)	C21—C22—H22	121.0
C5—C6—C7	122.40 (19)	C23—C22—H22	121.0
N12—C7—C8	121.14 (19)	N24—C23—C22	123.1 (2)
N12—C7—C6	116.98 (17)	N24—C23—H23	118.4
C8—C7—C6	121.88 (19)	C22—C23—H23	118.4
C9—C8—C7	119.1 (2)	C19—N24—C23	118.77 (17)
C9—C8—H8	120.4	C19—N24—Cd	118.43 (12)
C7—C8—H8	120.4	C23—N24—Cd	122.69 (14)
C10—C9—C8	119.5 (2)	C26—N25—Cd	152.39 (18)
C10—C9—H9	120.2	N25—C26—N27	173.1 (2)
C8—C9—H9	120.2	C26—N27—C28	122.02 (19)
C9—C10—C11	118.8 (2)	N29—C28—N27	172.9 (2)
C9—C10—H10	120.6	C31—N30—Cd	155.35 (19)
C11—C10—H10	120.6	N30—C31—N32	171.7 (2)
N12—C11—C10	122.0 (2)	C31—N32—C33	122.00 (19)
N12—C11—H11	119.0	N34—C33—N32	172.3 (2)
C10—C11—H11	119.0		
